# Characterization of the non-stationary nature of steady-state visual evoked potentials using echo state networks

**DOI:** 10.1371/journal.pone.0218771

**Published:** 2019-07-05

**Authors:** David Ibáñez-Soria, Aureli Soria-Frisch, Jordi Garcia-Ojalvo, Giulio Ruffini

**Affiliations:** 1 Neuroscience Research Business Unit, Starlab Barcelona S.L., Barcelona, Spain; 2 Department of Experimental and Health Sciences, Universitat Pompeu Fabra, Barcelona, Spain; 3 Neuroelectrics Corporation, Cambridge, Massachusetts, Unites States of America; Georgia State University, UNITED STATES

## Abstract

State Visual Evoked Potentials (SSVEPs) arise from a resonance phenomenon in the visual cortex that is produced by a repetitive visual stimulus. SSVEPs have long been considered a steady-state response resulting from purely oscillatory components phase locked with the stimulation source, matching the stimulation frequency and its harmonics. Here we explore the dynamical character of the SSVEP response by proposing a novel non-stationary methodology for SSVEP detection based on an ensemble of Echo State Networks (ESN). The performance of this dynamical approach is compared to stationary canonical correlation analysis (CCA) for the detection of 6 visual stimulation frequencies ranging from 12 to 22 Hz. ESN-based methodology outperformed CCA, achieving an average information transfer rate of 47 bits/minute when simulating a BCI system of 6 degrees of freedom. However, for some subjects and stimulation frequencies the detection accuracy of CCA exceeds that of ESN. The comparison suggests that each methodology captures different features of the SSVEP response: while CCA captures purely stationary patterns, the ESN-based approach presented here is capable of detecting the non-stationary nature of the SSVEP.

## 1. Introduction

Steady-state visual evoked potentials (SSVEPs) are a neural phenomenon that appears when neural oscillatory activity, mainly in the primary visual cortex, synchronizes to a repetitive visual light source. It can be measured in the EEG as oscillatory components that match the stimulation frequency and/or its harmonics [1]. Specifically, the SSVEP response is characterized by an energy increase that is phase-locked with the visual stimulus and is elicited for stimulation frequencies in the 1 to 100 Hz range [2]. The characteristics of the SSVEP response, mainly the amplitude and latency, show large inter-subject variations [3, 4, 5] and are also influenced by the stimulus source, its frequency, intensity, color, and duty cycle [1].

SSVEPs have proved to be useful for many paradigms in cognitive and clinical neuroscience [6]. For example, they are widely used for the characterization of spatial selective attention [7], and recent studies correlate SSVEP patterns with age-related memory performance loss [8,9]. In [10] a reduction of frontal SSVEP amplitude and latency was observed in older adults, indicating an age decline of neural processes. Response to repetitive visual stimulation has also been applied to the characterization of neurodegenerative and psychiatric disorders. Abnormal frontal SSVEP activity has been reported in patients diagnosed from schizophrenia [11], and a reduction of the second harmonic response over the right occipital has been observed in children suffering from Autism Spectrum Disorders [12].

Recent years have also witnessed an increased interest in the use of steady state visual evoked potentials (SSVEPs) in brain computer interface (BCI) systems [1,13]. SSVEP-based BCIs offer an excellent signal to noise ratio, have a large information transfer rate, and require a short calibration time [14]. A typical SSVEP-based BCI system with *N*_*s*_ degrees of freedom employs *N*_*s*_ independent light sources flickering at different frequencies. Each light source is associated then to a particular action of the BCI system: when the user wants the system to perform a specific action, he\she shall gaze at its associated light source.

Many techniques are used for SSVEP characterization [15], among them Power Spectrum Density Analysis (PSDA), Minimum Energy Combination [16] and Canonical Correlation Analysis (CCA) [17]. All these techniques exploit the steadiness of the SSVEP response. CCA is nowadays a popular approach for analyzing SSVEPs, its performance and robustness has been proved to be higher than other traditional techniques [18].

The brain is a complex, dynamical system that generates non-stationary high dimensional EEG patterns [19]. This complexity limits in general the efficacy of non-dynamical feature extraction and classification methods. Non-stationary techniques have been rarely applied to SSVEP analysis, due to the assumed stationary nature of the SSVEP response. In this work we aim to investigate the dynamical characteristics of SSVEP response using Recurrent Neural Networks, in their Reservoir Computing flavor. Specifically, we hereby propose a novel approach for extraction and classification of SSVEP features that uses an ensemble of Echo State Networks. The performance of the proposed approach is compared to that of standard canonical correlation analysis, which is believed to capture the stationary nature of the SSVEP response. A BCI system with six degrees of freedom has been implemented experimentally with stimulation frequencies ranging from 12 to 22 Hz, and the detection accuracy of each approach has been used as performance measure.

This paper is structured as follows. In section 2 we provide an overview of reservoir computing. We present the new methodology based on ESN in section 3 and introduce CCA. In section 4 we describe our experimental protocol, and in section 5 we provide results for both stationary and dynamical approaches. We conclude with a discussion of the results in section 6.

## 2. Reservoir computing

Artificial neural networks have been extensively used for the analysis of stationary problems in computational intelligence. These architectures are well understood due to their feed-forward structure and non-dynamical nature. It is in general not possible to detect temporal dynamics using feedforward structures. A possible temporal generalization strategy is to add recurrent connections, which allow the system to encode time-dependent information by providing the network with memory and transforming it into a dynamical system [20, 21]. Dynamical systems are commonly used to model non-stationary physical phenomena. These models are extensively used in a wide variety of fields including finance [22], economics [23] and physiology [24].

Since the early 1980s, a wide variety of approaches for adaptive learning in networks with recurrent connections have been proposed [25]. Training RNNs has traditionally been more complex and computationally more expensive than training feedforward neural networks. Additionally, cyclic connections can undergo bifurcations that lead to drastic changes in the system’s behavior [26]. Echo State Networks (ESN) [27] and Liquid State Machines [28] constitute an approach towards training and applying Recurrent Neural Networks, grouped together under what has come to be known as reservoir computing (RC). ESN is based on the principle that supervised adaptation of all interconnection weights in RNNs is not necessary if the untrained weights, known as the dynamical reservoir, fulfills certain algebraic properties [29], and thus significantly few number of connections need to be trained. This approach has certain analogies with kernel methods in machine learning, with the reservoir performing a nonlinear high-dimensional projection of the input signal for discriminating samples that are not linearly separable in the original space, and at the same time, serving as a memory that provides the temporal context [30].

In previous works we have demonstrated that among other dynamics ESNs can detect chaotic synchronization variations within temporal series [31]. Reservoir computing, and in particular ESNs, have been successfully applied in the past for EEG feature extraction and classification. In brain computer interfaces, ESNs were used in P300-based [32] and motor imagery [33] modalities, but never to our knowledge for SSVEP characterization. In the field of EEG biomarker discovery, ESNs have been employed to characterize abnormal EEG patterns in children diagnosed of Attention Deficit Hyperactivity Disorder (ADHD) [34] and Parkinson Disease (PD) patients [35] as well as for epileptic seizure detection [36].

**Fig 1:**
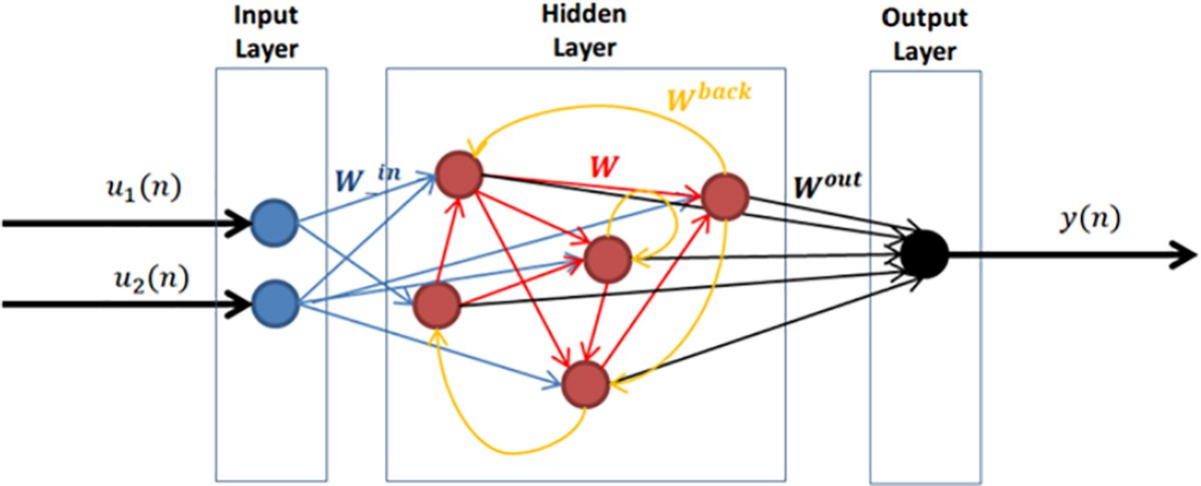
Recurrent neural network structure configured with 2 input units and 1 output unit (K = 2 and L = 1) and 5 internal units (N = 5).

The global structure of an RNN is depicted in Fig 1. Following the nomenclature and model used by Jaeger in [21], we consider a network of *K* input units, N internal units, and L output units. Input, internal and output connection weights are defined respectively by the connection weight matrices *W*^*in*^, *W* and *W*^*out*^. RNNs are characterized by having cyclic paths of connections defined by the back-projection weight matrix *W*^*back*^. Input (*W*^*in*^), internal (*W*) and back-propagation weights (*W*^*back*^) matrices conform the dynamical reservoir (DR) and are randomly constructed. The weight matrix *W* is characterized by its spectral radius, defined as the largest absolute eigenvalue of the weight matrix. The spectral radius is closely connected with the intrinsic dynamical timescale of the reservoir, and is therefore a key ESN training parameter. A small spectral radius leads to a faster RNN response.

For the ESN principle to work, the reservoir must fulfill the echo state property (ESP). The ESP holds if the current state of the network, which is running for an infinite time, is uniquely determined by the history of the input and the teacher-forced output (i.e. the initial state of the RNN does not matter, since it is forgotten). The echo state property has proved to be linked with the characteristics of the reservoir, with the input signals and with the input and back-propagation weights [37, 38, 39]. In most practical applications, a spectral radius below unity ensures the echo state property [40], although this is not a sufficient condition.

Other key training parameters in RC are the input scaling and the model size [40]. The model size is defined by the number of internal units N. Generally, a larger DR can learn more complex dynamics. It is very important however to be aware of the possible over-fitting if a large number of internal units is chosen, which would lead to poor generalization. The input scaling determines the degree of nonlinearity in the reservoir responses. Tasks close to linear require small input scaling factors, while highly nonlinear tasks demand larger input scaling values.

When constructing an ESN the first step is thus the random dynamical reservoir (DR) construction for the selected global parameterization. Its role is to provide temporal context and work as a nonlinear expansion of the input. The DR random and feedback connections guarantee different dynamics or states to echo within the reservoir. The larger the reservoir is, the richer its dynamics. That is why the number of units in ESNs is larger compared to other recurrent neural network architectures. During the training process the input signal may provoke dynamics that follow certain trajectories within the randomly constructed DR. ESNs training is a supervised machine learning problem in which for a training input and target output, the output weights of the RNN linearly combine these trajectories to minimize the error between the network output and the desired target. Output weights can be calculated using any linear regression algorithm.

## 3. Methods

### *3*.*1*. Stationary SSVEP detection

Canonical correlation analysis [41] is employed to evaluate the stationary characteristics of the SSVEP response. CCA is a multivariable calibration-less statistical method to calculate the maximal correlation between two multi-channel signals. CCA is widely used in statistical analysis and information mining [42,43]. SSVEP-based systems have largely used CCA-based methods in recent years due to its excellent accuracy and information transfer rate [44,45]. Given two multidimensional random variables *X*, *Y* and their linear transformation $\tilde{x}=w^{T}X$ and $\tilde{y}=v^{T}Y$, CCA finds the weight vectors *w* and *v* that maximize the correlation between $\tilde{x}$ and $\tilde{y}$. Canonical correlation therefore seeks a pair of linear transformations for *X* and *Y* such that when the multidimensional variables are transformed, the corresponding coordinates are maximally correlated [46]:

$$\rho =max\frac{E[\tilde{x}\tilde{y}]}{\sqrt{E[{\tilde{x}}^{2}]E[{\tilde{y}}^{2}]}}=\frac{w^{T}XYv}{\sqrt{w^{T}XXwv^{T}YYv}}$$ (1)

The SSVEP response is characterized by oscillations in the visual cortex matching the stimulation frequency and its harmonics. The performance of a given stimulation frequency *f*_*i*_ is evaluated by computing the canonical correlation between the EEG sequence under evaluation (*X*) and a reference signal (*Y*), constructed as a set of sine-cosine series at the stimulation frequency and its *N*_*h*_ harmonics of duration equal to that of the EEG sequence:

$$Y=\left(\begin{array}{c} \sin(2\pi f_{i}t)\\ Cos(2\pi f_{i}t)\\ \ldots \\ sin(2\pi N_{h}\;f_{i}t)\\ Cos(2\pi N_{h}\;f_{i}t) \end{array}\right)$$ (2)

The maximal canonical correlation (*ρ*_*i*_) is calculated for all *N* stimulation frequencies (*f*_*i*_) under test. The stimulation frequency delivering the largest canonical correlation (*ρ*) is selected as responsible of eliciting the visual response [47].

### *3*.*2*. Dynamical SSVEP detection

A novel approach for extraction and classification of SSVEP non-stationary patterns is proposed here. This approach is based on an ensemble of as many echo state networks as stimulation frequencies under test. Each ESN is therefore responsible of detecting the elicited response of a particular flickering frequency.

#### *3*.*2*.*1*. Temporal SSVEP feature extraction

SSVEP EEG temporal components are calculated for each of the *N*_*e*_ electrode signals (which are denoted as *x*_*i*_(*n*), *i* = 1,2,3…*N*_*e*_). The SSVEP temporal response at *f*_*i*_ for the harmonic *K* is computed by filtering the raw EEG using a narrow band-pass FIR filter with its central frequency at *K*∙*f*_*i*_. The ensemble temporal response ($x_{i}^{f_{i}}(n)$) is computed by adding the calculated individual response at every calculated harmonic. FIR filters were selected as they present linear phase. Their induced delay is thus the same at all frequencies not causing phase or delay distortion. They are inherently stable since they have no feedback elements. The EEG signal was feed-forward filtered and then the fixed delay introduced by the filtering corrected. Filters were designed using a Hamming window using the Matlab fir1 function. The order of filter was 501 coefficients. Bandpass filter cut-off frequencies were set at 0.5 Hz above and below the target frequency.

#### *3*.*2*.*2*. ESN construction

**Fig 2:**
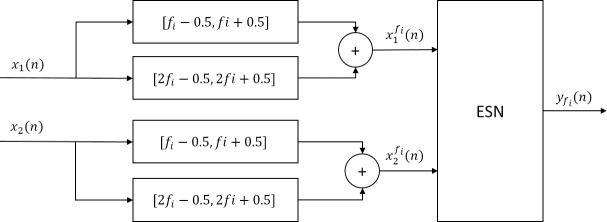
Proposed RC-based SSVEP feature extraction architecture for detection of the stimulation frequency *f*_*i*_ using up to the second harmonic and *N*_*e*_ = 2.

ESNs (one per stimulation frequency) have been configured to have as many input nodes as there are EEG electrodes that measure the visual evoked response (*N*_*e*_). As will be explained in following sections, the optimal number of internal units, spectral radius, and input scaling factor has been selected by using three-fold cross validation exhaustive search. The temporal decomposition of SSVEP components coming from each electrode ($x_{i}^{f_{i}}(n)$)) feeds the *N*_*e*_ input nodes of the ESN targeting detection of *f*_*i*_. The proposed ESN detection and classification methodology is applied to an EEG recording acquired during the interleaving of *N*_*s*_ non-stimulation periods followed by *N*_*s*_ stimulation ones, where the visual stimulation is presented at *f*_*i*_. The function of the ESN is to discriminate visual stimulation periods at *f*_*i*_ from non-stimulation periods or stimulation periods at other frequencies. For this two-class regression problem, during ESN training, the network outputs ($y_{f_{i}}(n)$) of samples corresponding to stimulation periods at *f*_*i*_ are set to 1, while the output of samples corresponding to non-stimulation periods are set to -1. Therefore, during the ESN recall the associated ESN output is maximized during the visual stimulation period. Fig 2. depicts the architecture for the characterization of *f*_*i*_ with *N*_*e*_ = 2 and *N*_*h*_ = 2. It is important to recall, that while being trained on binary labels, the ESN output is continuous.

#### *3*.*2*.*3*. Stimulation frequency detection

SSVEP frequency detection aims at determining which stimulation frequency among the ones under evaluation (*f*_*i*_ with *i* = 1,2…*N*) elicited the visual response during a stimulation trial. To achieve this, the ESN-based architecture has been designed as an ensemble of *N* ESNs, where each ESN has been trained for each stimulation frequency *f*_*i*_. The SSVEP response at each frequency ($R_{f_{i}}$) is assessed as the difference between the averaged ESN output ($y_{f_{i}}(n)$) during the stimulation observation window and a baseline sequence prior the stimulation. As the ESN output is maximized during stimulation periods at the trained stimulation frequency, the stimulation frequency with maximal $R_{f_{i}}$ will be selected as responsible of eliciting the visual response.

## 4. Experimental method

Five Caucasian male volunteers S1 to S5 with average age 33.6 years, and ages ranging from 29 to 46, participated in six recording sessions each, where oscillatory visual stimuli were presented at six different frequencies *f*_*i*_: 12, 14, 16, 18, 20 and 22 Hz. The visual stimuli were presented using stimulation sources consisting of an array of flickering light emitting diodes (LEDs) through a diffusing panel of 100 squared centimeters. Neuroelectric’s regulatory department acting as ethics committee stated that this experimental campaign was exempt from ethical review. The electroencephalographic recordings were performed on healthy adults. Neither the recording technique, nor the experimental protocol deemed to entail any danger or discomfort for the participants. Participant’s enrolled were professionals working in the field of neuroscience, who were fully aware of the recording technique and protocol. Consequently only oral informed consent was only required. Participants invited to take part in this study received detailed information of the experimental procedure before the session. This explanation was provided by the technician in charge of performing the recording. They were informed that 1) they will undertake periodic visual stimulation, 2) EEG sensors will be placed on their head and conductive gel inserted between the sensors and the scalp in order to ensure adequate contact, 3) a remote possibility of a mild headache and\or skin irritation, 4) that the study aimed to analyze alterations in brain patterns induced by a visual stimulation, and 5) they could quit the experiment at any time if they so desire. They had the opportunity to ask the technician to clarify anything they did not understand or would like to know more about. Participants confirmed they had understood the explanations, and that they voluntarily engage in the study. The researcher responsible of performing the experiment documented this consent in a signed document in which for each participant the date, time and location where the experiment took place were recorded. In this document the researcher declared to have properly informed the participant and that he/she voluntarily agreed to take part in this study.

Each session consisted of one recording per stimulation frequency. In each recording, *N*_*s*_ = 15 stimulation trials (duration randomly ranging from 4 to 5 seconds), where the visual stimulus was presented, were followed by the same number of non-stimulation trials (duration randomly ranging from 5 to 8 seconds) with no visual stimulation. One stimulation source placed on the right of the subject presented the stimulation frequency under evaluation, while another source on the left presented a frequency randomly selected among the other frequencies used in the experiment. The goal of this montage was to simulate background interferences as in brain computer interfaces applications. Stimulation sources were separated by approximately 25 cm.

The user was comfortably seated at one-meter distance from the stimulation sources and was instructed to look at the stimulation source placed on his right when hearing a beep sound (played one second before the stimulation started). EEG was acquired using Neuroelectrics Enobio, a wearable, wireless electrophysiology sensor system for the recording of EEG, at a sampling rate of 250Hz and from three channels placed in O1, Oz and O2, according to the 10–20 system [48]. The electrical reference was placed in the right ear-lobe. Background ambient light remained homogeneous throughout all experimental sessions. The AsTeRICS [49] platform was used to record the EEG streaming data, control the stimulation panels, and trigger the recording.

## 5. Performance evaluation and results

The goal of the performance evaluation is to compare how well the SSVEP response is detected using stationary and dynamical approaches. Using previously presented ESN and CCA based methods, the stimulation frequency among the 6 under evaluation (12, 14, 16, 18, 20 and 22 Hz) responsible for eliciting the evoked potential at each trial is assessed. The performance will be estimated for observation windows ranging from 0.5 to 4 seconds in terms of detection accuracy and information transfer rate (ITR) [50].

### *5*.*1* ESN based approach

#### *5*.*1*.*1* ESN parameterization

ESN networks have been configured to have *N*_*e*_ = 3 input nodes and one output node. Input nodes are fed with the filtered signals coming from O1, Oz and O2. The activation function of the network nodes is set to a hyperbolic tangent. Least-mean-square error minimization between the network output and the target output has been used to compute output weights. A washout duration of 250 samples has been applied. The optimal number of internal units, spectral radius and input scaling has been calculated through exhaustive search. The detection accuracy calculated over 4-second observation windows and a 0.5-second baseline has been assessed for every combination of internal units (10, 50, 100, 150, 200, 250 and 300), spectral radius (from 0 to 1 in steps of 0.1) and input scaling factor (0.001, 0.005, 0.01 0.05 0.1 and 1). A total of 462 combinations were attempted.

Data is split into a training and a test set. The training set consists of the first 10 stimulation sequences, while the test set contains the remaining 5. The training set is used to calculate the optimal ESN parameterization, whereas the test set is used to evaluate the performance of the final model parameters. Parameter optimization was done based on 5 times 2-fold-cross validation (5 x 2-FCV). The cross-validation process was repeated 5 times in order minimize the effect introduced by the random dynamical reservoir initialization. In this process, the training set is partitioned in two non-overlapping folds for independently calculating the readout connections of the ESN and assessing the model under evaluation performance. Each fold is formed by 5 sequences of a stimulation trial (duration randomly ranging from 4 to 5 seconds), where the visual stimulus was presented, followed by a non-stimulation trial (duration randomly ranging from 5 to 8 seconds). The average fold duration was therefore 55 seconds (13750 samples).

Parameter optimization was done through grid search for every possible combination of the spectral radius, input scaling and network size values. In each stimulation sequence under evaluation, the frequency maximizing the difference between the average ESN output in the 4-second observation window and the average ESN output in a 0.5-second baseline measured right before the stimulation trial is selected as responsible of eliciting the visual evoked response. A spectral radius of 0.5, 250 internal units and 0.05 input scaling factor delivered the best average classification accuracy across subjects (66%).

#### *5*.*1*.*2* RC-ESN SSVEP detection

**Fig 3:**
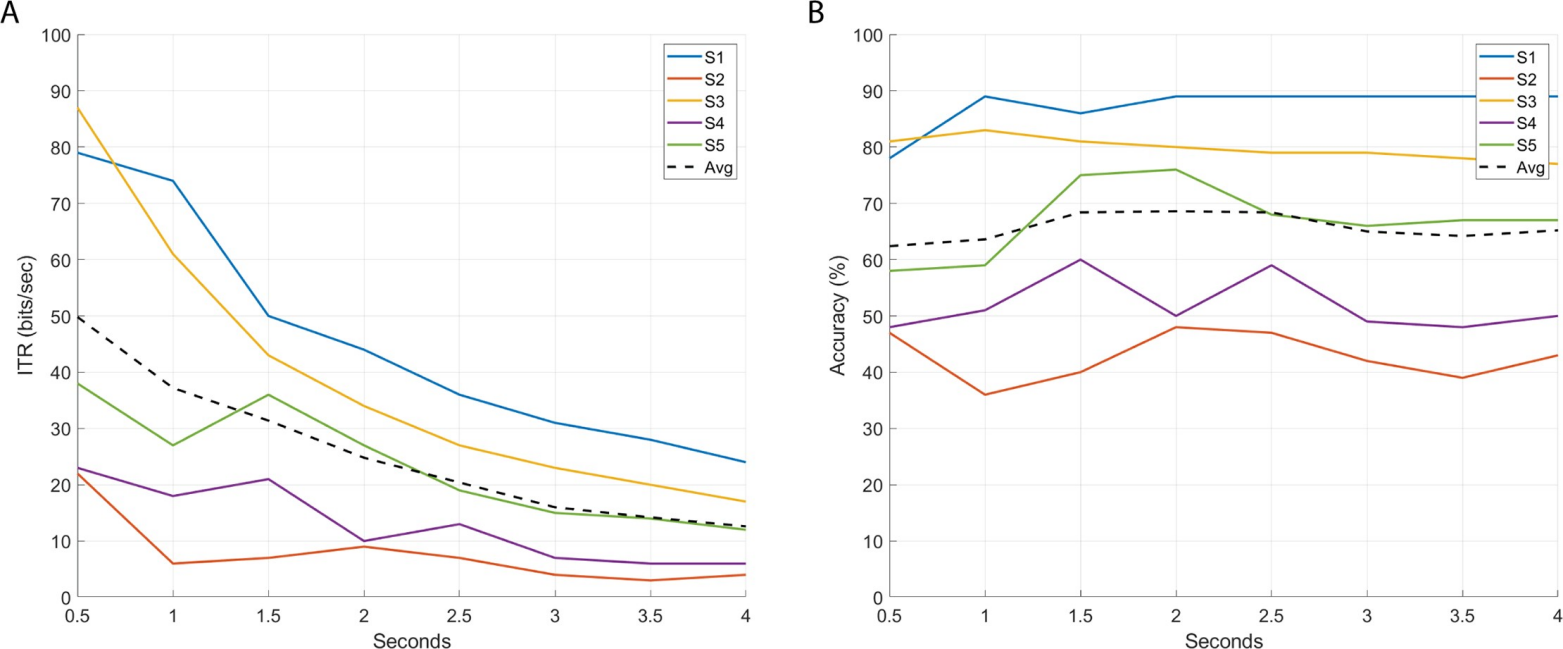
Information transfer rate (A) and detection accuracy (B) at different observation-window length using Echo State Networks detection.

Final results were calculated for the parameters that delivered the best average classification accuracy in Section 5.1.1, namely a spectral radius of 0.5, 250 internal units and 0.05 input scaling factor, that are applied to SSVEP detection in 0.5, 1, 1.5, 2, 2.5, 3, 3.5, and 4-second observation windows using a baseline of 0.5 seconds. The test set as described in Section 5.1.1, consisting of the 5 stimulation sequences not used for the ESN global parameters search, is employed to evaluate the ESN detection performance at each observation window. The average of five independent iterations has been calculated in order to reduce the effect produced by the random dynamical reservoir construction. Table 1 presents the calculated detection accuracy and ITR, considering the trial duration as the addition of the observation window and the initial baseline (0.5-seconds). Fig 3A and 3B show respectively individual ITR and detection accuracy for each participant. The observation window duration does not influence the detection accuracy of the RC-ESN-based detection. Similar average and individual detection performance is achieved for every observation window length under test. This fact boosts the maximum obtained ITR that reaches 49 bits/minute in 0.5-second windows. An important subject variability is observed, while some subjects achieve an excellent communication performance (Subject 3 has an ITR of 87 bits/min in 0.5-second windows), in others it significantly decreases (ITR for subject 2 is 22 bits per minute).

**Table 1:** Detection accuracy percentage and ITR (within brackets) in bits/minute.

	0.5”		1”		1.5”		2”		2.5”		3”		3.5”		4”	
	cca	esn	cca	esn	cca	esn	cca	esn	cca	esn	cca	esn	cca	esn	cca	esn
**S1**	17 (0)	78 (79)	27 (3)	89 (74)	47 (14)	86 (50)	67 (27)	89 (44)	70 (24)	89 (36)	63 (16)	89 (31)	77 (22)	89 (28)	73 (17)	89 (24)
**S2**	10 (0)	47 (22)	23 (1)	36 (6)	30 (3)	40 (7)	40 (7)	48 (9)	50 (10)	47 (7)	53 (10)	42 (4)	57 (10)	39 (3)	63 (12)	43 (4)
**S3**	23 (2)	81 (87)	63 (47)	83 (61)	90 (75)	81 (43)	90 (57)	80 (34)	97 (56)	79 (27)	97 (46)	79 (23)	97 (40)	78 (20)	100 (39)	77 (17)
**S4**	17 (0)	48 (23)	17 (0)	51 (18)	23 (1)	60 (21)	33 (3)	50 (10)	37 (4)	59 (13)	50 (8)	49 (7)	53 (9)	48 (6)	67 (14)	50 (6)
**S5**	20 (1)	58 (38)	27 (3)	59 (27)	23 (1)	75 (36)	23 (1)	73 (27)	20 (0)	68 (19)	20 (0)	66 (15)	27 (1)	67 (14)	27 (1)	67 (12)
**Avg**	17 (0)	63 (47)	31 (5)	64 (32)	43 (11)	68 (28)	51 (13)	68 (23)	55 (13)	68 (19)	57 (12)	65 (14)	62 (13)	64 (12)	66 (13)	65 (11)

### *5*.*2* Canonical correlation analysis

Recordings have been band-pass filtered using a finite impulse response filter with 250 coefficients and high and low cut-off frequencies set to 1 and 45 Hz, respectively. The filtering aims to reduce the influence of high amplitude low-frequency components caused by motion artifacts and bad electrode-skin contact, as well as removing power line-noise interferences. After filtering, stimulation sequences have been extracted and split into shorter observation windows starting at the beginning of the visual stimulation. Maximal canonical correlation has been calculated for each stimulation frequency and its second harmonic. Canonical correlation performance has been evaluated on the test set used to assess the performance of the final ESN model.

**Fig 4:**
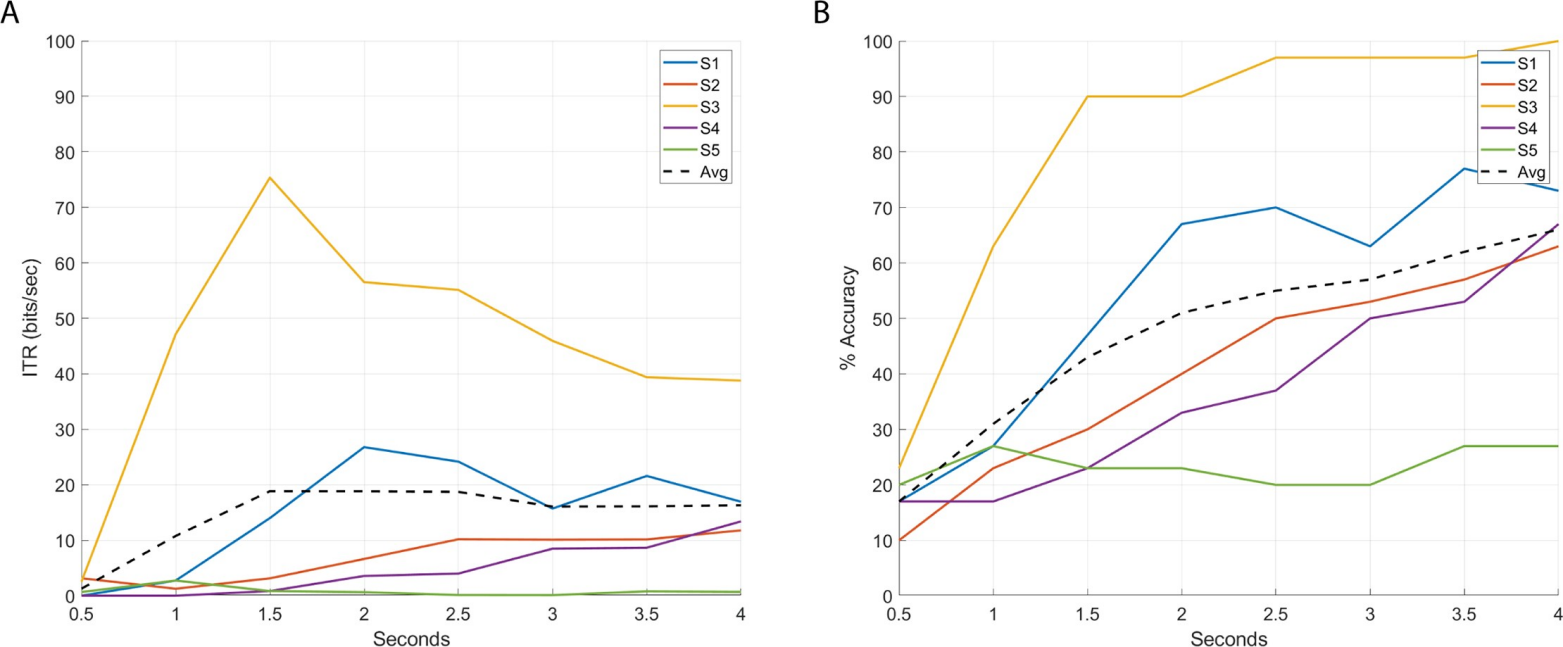
Information transfer rate (A) and detection accuracy (B) at different observation-window length using standard CCA detection.

Table 1 presents the detection accuracy and ITR for 0.5, 1, 1.5, 2, 2.5, 3, 3.5, and 4-second observation windows. Fig 4A and 4B displays respectively the individual ITR and detection accuracy for each participant for the proposed observation windows. Detection accuracy increases along with the observation window length, achieving a maximum average of 66% in the 4-second observation window. The maximum information transfer rate, 13 bits/minute, is reached at 2-second observation windows.

The data shows an important amount of subject variability: while some subjects achieve an excellent detection accuracy and ITR (Subject 3 has 100% classification accuracy for a 4-second observation window and a ITR of 75 bits/minute in 1.5-second windows), others deliver poor classification results (Subject 5 has 27% detection accuracy for 4-second observation windows and a maximum ITR of 3 bits/minute).

### *5*.*3* Stationary Vs dynamical SSVEP frequency detection

SSVEP is a subject-dependent phenomenon in which a given stimulation frequency has proved to range from excellent to random classification across subjects [36]. Table 2 presents the detection accuracy for each stimulation frequency obtained for the observation windows delivering largest average detection accuracy for CCA and ESN-based methodologies, respectively 4 and 2 seconds.

**Table 2:** Detection accuracy percentage of canonical correlation analysis (4-seconds observation window) and proposed ESN-based methodologies (2-seconds observation window). In red accuracies below 50%, in green in the 50–75% range and in green above 75%.

	Subject 1		Subject 2		Subject 3		Subject 4		Subject 5	
	cca w = 4”	esn w = 1.5”	cca w = 4”	esn w = 1.5”	cca w = 4”	esn w = 1.5”	cca w = 4”	esn w = 1.5”	cca w = 4’	esn w = 1.5”
**12Hz**	100	100	100	60	100	16	60	4	80	88
**14Hz**	80	80	80	24	100	100	100	20	40	64
**16Hz**	100	100	80	12	100	100	100	76	0	36
**18Hz**	80	72	40	40	100	68	80	72	0	72
**20Hz**	40	80	20	36	100	100	40	96	40	80
**22Hz**	40	100	60	80	100	100	20	60	0	80
**Avg**	**73**	**89**	**63**	**42**	**100**	**81**	**67**	**55**	**27**	**70**

ESN-based methodologies significantly improve the average detection accuracy of subjects 1 and 5, while standard CCA performs better in subject 2 and 4. Both methodologies deliver excellent average classification performance for subject 3. Results show that the ESN-based method significantly improves classification in some stimulation frequencies and subjects compared to CCA (subject 5, stimulation frequency 22 Hz) and vice versa (subject 2 stimulation frequency 14 Hz).

## 6 Discussion and conclusions

We have presented a novel SSVEP-detection methodology based on reservoir computing with non-stationary pattern recognition capabilities whose performance has been compared with state of the art SSVEP stationary detection, Canonical Correlation Analysis. In this study the response to 6 visual stimulation frequencies ranging from 10 to 22 Hz is studied in 5 participants. Stationary and dynamical analysis of SSVEP response delivered disparate classification accuracy as displayed in Table 2. While for some subjects ESN-based methodologies outperformed the stationary analysis in terms of detection accuracy (Subject 5 at 22Hz) in others the opposite occurred (Subject 2 at 16Hz). Our hypothesis is that this performance may prove the elicitation of evoked responses of different nature, explaining why stationary and dynamical detection methodologies behave differently.

Traditionally brain response to a flickering visual stimulation has been considered steady-state, in which the elicited effect is believed to be unchanging in time. In this scenario CCA can capture this stationary nature by linearly transforming the EEG into a vector space that reproduces the flickering frequency and its harmonics. In contrast, as previously stated in Section 2, Reservoir Computing based approaches can detect dynamical patterns and complex synchronization between EEG channels, and therefore identify the non-stationary nature of the SSVEP. The study of this SSVEP response duality can be used to further understand brain mechanisms in cognitive and clinical neuroscience. Our hypothesis is that SSVEP dynamical response may serve to study alterations in brain patterns linked attention, aging neurodegeneration and psychiatric disorders.

The hereby proposed methodology can be also employed by itself or combined with other stationary techniques for the construction of BCI applications. Its detection capabilities proved to be independent from the observation window duration, delivering similar detection accuracy for windows ranging from 0.5 to 4 seconds (from 63% to 68%). In contrast, in CCA detection, performance increases along with window length, starting at random classification for 0.5-second windows and reaching a detection accuracy of 66% in 4-second windows. These results highlight the communication capabilities of the ESN-based method, which achieves an average information transfer rate of 47 bits/minute (with a maximum ITR of 87 bits/minute for Subect 3), that outperforms the average information transfer rate of 13 bits/minute achieved by the CCA method. The methodology proposed here achieves a high communication rate for a BCI system with 6 degrees of freedom, compared to other state of the art applications [1, 51, 52]. However, given the reduced data set of this study, although training-test split methodology provides a reasonable sanity check, the low number of samples reduce the ability of this statistical test to robustly generalize. The applicability of the here proposed ESN-based methodology in BCI systems needs therefore to be further explored on larger data sets.
